# Crystal structure of di­ethano­lbis(thio­cyanato)­bis(urotropine)cobalt(II) and tetra­ethano­lbis(thio­cyanato)­cobalt(II)–urotropine (1/2)

**DOI:** 10.1107/S2056989021013281

**Published:** 2022-01-01

**Authors:** Christoph Krebs, Inke Jess, Magdalena Ceglarska, Christian Näther

**Affiliations:** a Institute of Inorganic Chemistry, University of Kiel, Max-Eyth.-Str. 2, 24118 Kiel, Germany; b Institute of Physics, Jagiellonian University, Lojasiewicza 11, 30-348 Kraków, Poland

**Keywords:** crystal structure, discrete complexes, cobalt thio­cyanate, urotropine, hydrogen bonding

## Abstract

The reaction of one equivalent Co(NCS)_2_ with four equivalents of urotropine (hexa­methyl­ene­tetra­mine) in ethanol leads to the formation of two compounds, namely, bis­(ethanol-κ*O*)bis­(thio­cyanato-κ*N*)bis­(urotropine-κ*N*)cobalt(II), [Co(NCS)_2_(C_6_H_12_N_4_)_2_(C_2_H_6_O)_2_] (**1**), and tetra­kis­(ethanol-κ*O*)bis­(thio­cyanato-κ*N*)cobalt(II)–urotropine (1/2), [Co(NCS)_2_(C_2_H_6_O)_4_]·2C_6_H_12_N_4_ (**2**). In **1**, the Co cations are located on centers of inversion and are sixfold coordinated by two terminal N-bonded thio­cyanate anions, two ethanol and two urotropine ligands whereas in **2** the cobalt cations occupy position Wyckoff position *c* and are sixfold coordinated by two anionic ligands and four ethanol ligands. Compound **2** contains two additional urotropine solvate mol­ecules per formula unit, which are hydrogen bonded to the complexes. In both compounds, the building blocks are connected *via* inter­molecular O—H⋯N (**1** and **2**) and C—H⋯S (**1**) hydrogen bonding to form three-dimensional networks.

## Abstract 2

The crystal structure of both compounds consists of discrete complexes with terminal N-bonded thio­cyanate anions in which the Co cations are sixfold coordinated within slightly distorted octa­hedra, which are linked by inter­molecular hydrogen bonding, in one compound *via* additional urotropine solvate mol­ecules, into three-dimensional networks.

## Chemical context

Thio­cyanate anions are versatile ligands that exhibit a variety of coordination modes, leading to rich structural chemistry (Näther *et al.*, 2013 ▸). For less chalcophilic metal cations such as Mn^II^, Fe^II^, Co^II^ or Ni^II^, most compounds contain terminal N-bonded thio­cyanate anions, whereas for chalcophilic metal cations such as for example Cd^II^, the μ-1,3-bridging mode is preferred. Therefore, the synthesis of bridging compounds with the former cations is sometimes difficult to achieve, which is a pity, because such compounds are of inter­est due to their magnetic properties (Mautner *et al.*, 2018 ▸; Mekuimemba *et al.*, 2018 ▸; Mousavi *et al.*, 2020 ▸; Palion-Gazda *et al.*, 2015 ▸; Suckert *et al.*, 2016 ▸). This is especially the case with cobalt, which frequently exhibits inter­esting behavior due to its large magnetic anisotropy, so we and others have been studying such compounds for several years (Shi *et al.*, 2006 ▸; Jin *et al.*, 2007 ▸; Wellm *et al.*, 2020 ▸; Prananto *et al.*, 2017 ▸). Within this project we are inter­ested for example in the influence of the co-ligand on the magnetic anisotropy and the magnetic behavior of compounds, in which the cations are linked by thio­cyanate anions into chains (Böhme *et al.*, 2020 ▸; Rams *et al.*, 2020 ▸; Ceglarska *et al.*, 2021 ▸; Werner *et al.*, 2014 ▸, 2015 ▸).

In the course of our systematic work, we became inter­ested in urotropine as a co-ligand. Therefore, we reacted Co(NCS)_2_ with urotropine in aceto­nitrile, which leads to the formation of a compound with the composition [Co(NCS)_2_(H_2_O)_2_(urotropine)_2_]·(urotropine)_2_(MeCN)_2_ consisting of discrete complexes, which are linked by urotropine and aceto­nitrile solvate mol­ecules into a hydrogen-bonded network (Krebs *et al.*, 2021 ▸). In principle, the formation of discrete solvato complexes would be no problem because in several cases such complexes can be transformed by thermal decomposition into the desired compounds with a bridging coordination of the anionic ligands (Näther *et al.*, 2013 ▸), but XRPD measurements proved that this crystalline phase was not obtained pure.

In further work, we used ethanol as a solvent leading to the formation of two different crystals in the same batch that were characterized by single-crystal X-ray diffraction. The crystals in this batch were crushed and investigated by XRPD. Comparison of the experimental pattern with that calculated for **1** and **2** reveal that only **1** can be detected together with at least one additional and unknown crystalline phase. The reason for this observation is unclear, but it might be that **2** is unstable and transforms into a new phase on grinding. 
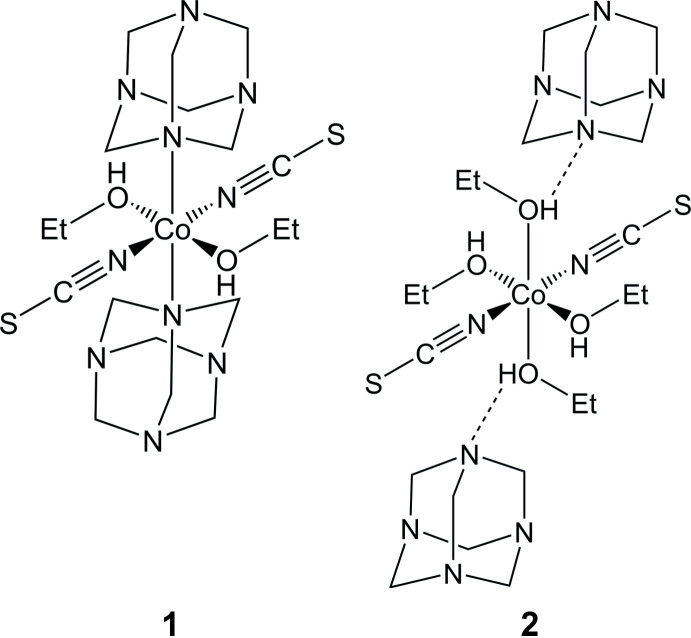


## Structural commentary

**Figure 1:**
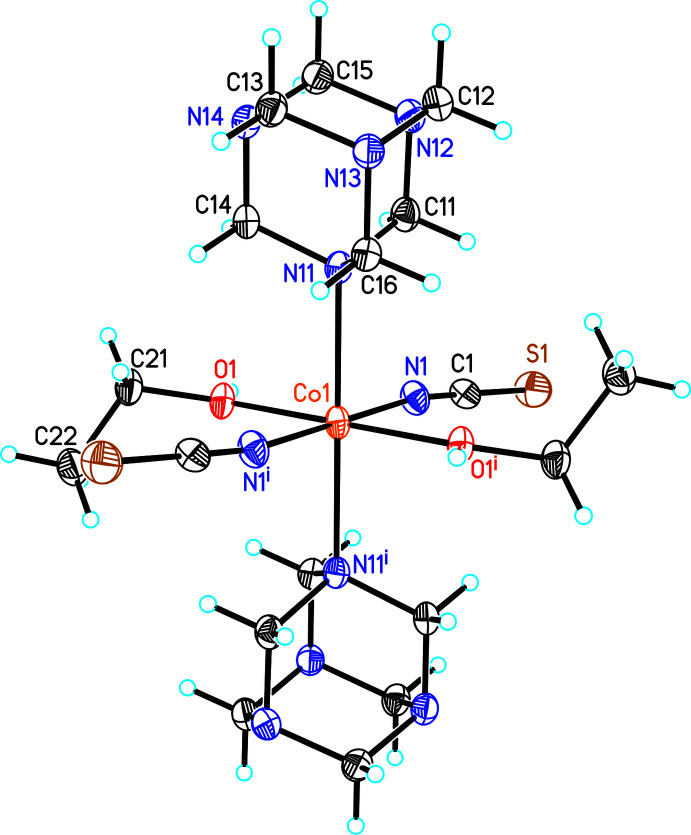
Crystal structure of compound **1** with labeling and displacement ellipsoids drawn at the 50% probability level. Symmetry codes for the generation of equivalent atoms: (i) −*x* + 1, −*y* + 1, −*z* + 1.

**Figure 2:**
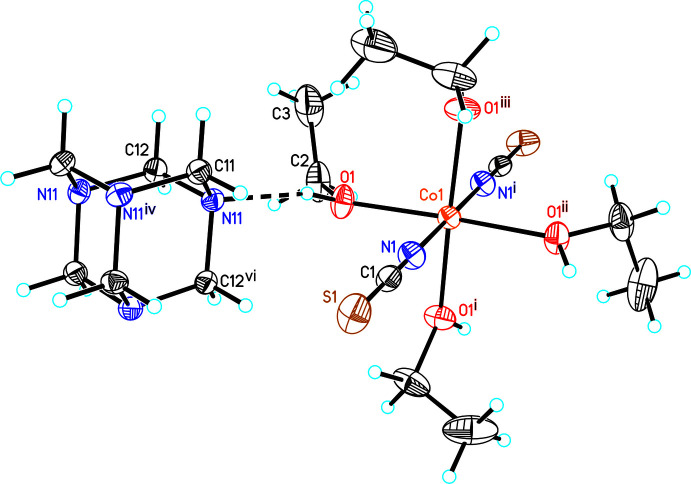
Crystal structure of compound **2** with labeling and displacement ellipsoids drawn at the 50% probability level with O—H⋯N hydrogen bonding shown as dashed lines. Symmetry codes for the generation of equivalent atoms: (i) −*x* − 1, −*y*, +*z*; (ii) *y* − 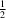 , 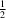 + *x*, *z* − 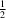 ; (iii) − 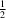 − *y*, − 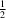 − *x*, − 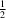 − *z*; (iv) 1 − *x*, 1 − *y*, +*z*; (v) *y*, −*x* − 1, −*z*; (vi) −*y* − 1, +*x*, −*z*.

The asymmetric unit of **1**, Co(NCS)_2_(urotropine)_2_(EtOH)_2_, consists of one crystallographically independent Co cation, located on a center of inversion, as well as one thio­cyanate anion, one urotropine ligand and one ethanol mol­ecule occupying general positions (Fig. 1 ▸). In **2**, [Co(NCS)_2_)(EtOH)_4_]·(urotropine)_2_, the asymmetric unit contains one cobalt cation on position of site symmetry 222 (Wyckoff position *c*), one thio­cyanate anion that is located on a twofold rotation axis and one urotropine mol­ecule on an inversion axis (Fig. 2 ▸). The Co—N distance to the thio­cyanate anions in **1** is slightly shorter than in **2**, whereas the Co—O bond length to the ethanol ligand is longer (compare Tables 1 ▸ and 2 ▸). The former can be traced back to the fact that in **2** the Co cation is exclusively coordinated by ethanol, whereas in **1** this cation is additionally coordinated by a urotropine ligand, which is a stronger donor than ethanol, transferring additional charge to the Co center. This leads to a strengthening of the Co—N thio­cyanate bond and therefore this bond length is shorter. This is also supported by previous investigations when discrete complexes with an N_6_ (four N atoms of N-donor co-ligands) or N_4_O_2_ (two N-donor co-ligands and two *e.g.* water mol­ecules) coordination were compared. For N_4_O_2_ coordin­ation, the CN stretching vibration of the thio­cyanate anions is significantly shifted to higher values, which indicates that the C—N bond becomes stronger, leading to a weakening of the Co—N bond (Böhme *et al.*, 2020 ▸). The angles around the Co cations deviate from the ideal octa­hedral values, which shows that the octa­hedra are slightly distorted (see supporting information). The octa­hedron in **2** is more distorted than in **1**, which is obvious from the octa­hedral angle variance (1.8138 for **1** and 8.1624 for **2**) and the mean octa­hedral quadratic elongation (1.0062 for **1** and 1.0023 for **2**) calculated by the method of Robinson *et al.* (1971 ▸).

## Supra­molecular features

**Figure 3:**
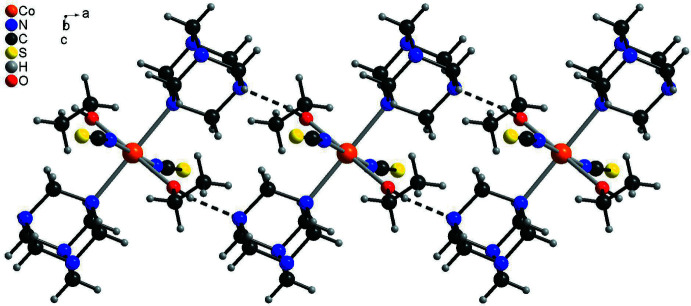
Crystal structure of compound **1** with a view of a chain formed by inter­molecular O—H⋯N hydrogen bonding along the crystallographic *a*-axis. Inter­molecular hydrogen bonding is shown as dashed lines.

**Figure 4:**
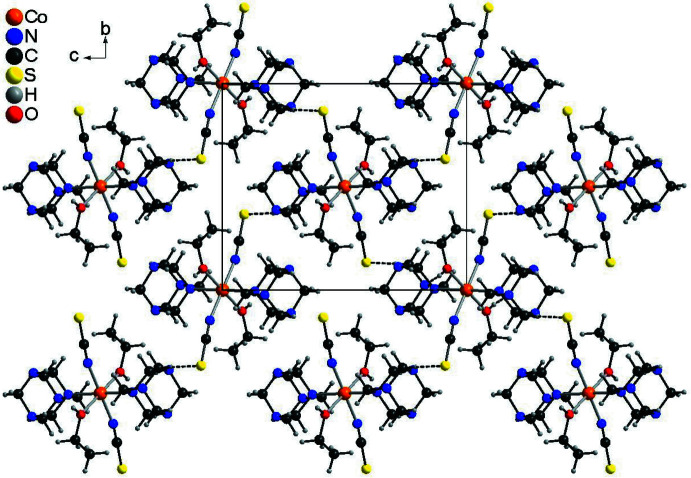
Crystal structure of compound **1** with a view along the crystallographic *a*-axis with inter­molecular C—H⋯S hydrogen bonding shown as dashed lines.

In the crystal structures of both compounds, inter­molecular hydrogen bonding is observed (Tables 3 ▸ and 4 ▸). In **1**, the discrete complexes are linked *via* inter­molecular O—H⋯N hydrogen bonding between the hydroxyl H atoms of one complex and the N atoms of neighboring complexes into chains extending in the *a*-axis direction (Fig. 3 ▸ and Table 3 ▸). These chains are further linked into a three-dimensional network by C—H⋯S hydrogen bonding between the thio­cyanate S atoms and each one H atom of urotropine ligands (Fig. 4 ▸). There are additional C—H⋯N and C—H⋯O contacts but from the distances and angles it is indicated that these are very weak inter­actions (Table 3 ▸).

**Figure 5:**
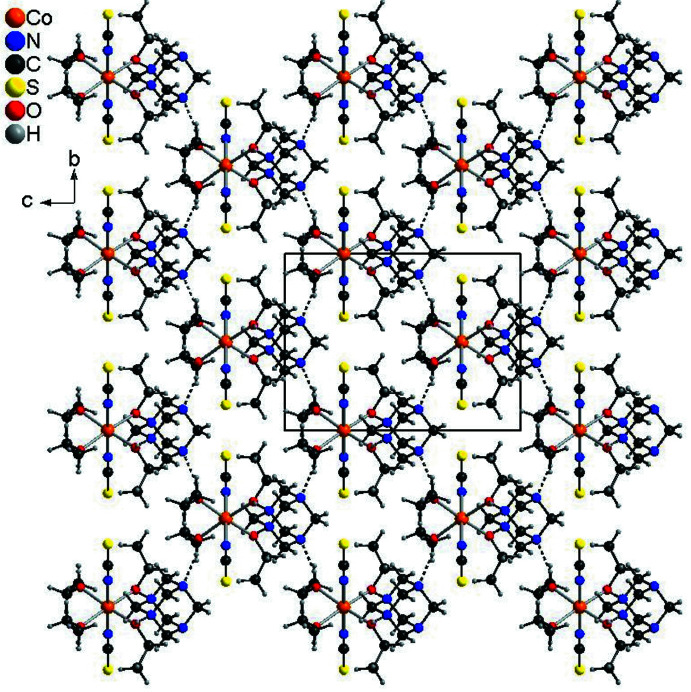
Crystal structure of compound **2** with a view along the crystallographic *a*-axis with inter­molecular O—H⋯N hydrogen bonding shown as dashed lines.

In the crystal structure of **2**, each complex is linked to neighboring complexes *via* inter­molecular O—H⋯N hydrogen bonds between the four O—H hydrogen atoms of one complex and the N atoms of the urotropine mol­ecules of four neighboring complexes to form a three-dimensional network (Fig. 5 ▸ and Table 4 ▸). From the H⋯N distance and the O—H⋯N angle it is obvious that this corresponds to a strong inter­action. In contrast to **1**, no C—H⋯S hydrogen bonding is observed and the additional C—H⋯N contact represents a weak inter­action (Table 4 ▸).

## Database survey

The Cambridge structure Database (CSD version 5.42, last update November 2020; Groom *et al.*, 2016 ▸) already contains some structures of transition-metal thio­cyanate coordination compounds with urotropine as a co-ligand. This includes a mixed complex with the composition [Co(NCS)_2_(C_6_H_12_N_4_)(CH_3_OH)_2_(H_2_O)], in which the cobalt cations are coordinated by water, ethanol, urotropine and N-bonded thio­cyanate anions (Refcode: POFGAT; Shang *et al.*, 2008 ▸). It also contains two compounds with the composition [Co(NCS)_2_(H_2_O)_4_]·2C_6_H_12_N_4_ (Refcode: XILXOG; Li *et al.*, 2007 ▸) and [Co(NCS)_2_(C_6_H_12_N_4_)_2_(H_2_O)_2_][Co(NCS)_2_(H_2_O)_4_]·2H_2_O (Refcode: MOTNIS; Liu *et al.*, 2002 ▸, MOTNIS01; Zhang *et al.*, 1999 ▸, MOTNIS02; Chakraborty *et al.*, 2006 ▸, MOTNIS03; Lu *et al.*, 2010 ▸), that also form discrete complexes with terminal N-bonded thio­cyanate anions. The structure of these compounds is somehow related to that in **1** and **2** with the major difference being that the ethanol is replaced by water. Discrete complexes have also been reported with other transition-metal thio­cyanates including, for example, nickel (Refcode: XILROA; Bai *et al.*, 2007 ▸, XILROA01; Lu *et al.*, 2010 ▸) and zinc (Refcode: SIMXIY; Kruszynski & Swiatkowski, 2018 ▸), but none of them contains ethanol as a co-ligand. The latter structure with the composition [Zn(NCS)_2_(urotropine)_2_(H_2_O)_2_]·[Zn(NCS)_2_(H_2_O)_4_]·2H_2_O contains two different complexes, one of them similar to **1** and the second similar to **2** with the difference that the EtOH is exchanged by water.

Finally, it is noted that with cadmium and mercury a crystal structure with urotropine is reported in which the Cd cations are linked by pairs of thio­cyanate anions into chains, which are further linked by the urotropine ligand (Refcode: DOZZOI; Bai *et al.*, 2009 ▸ and DIJSIY; Mak & Wu, 1985 ▸). The formation of such a compound can be traced back to the fact that cadmium and mercury are much more chalcophilic than cobalt. There is one additional structure with cadmium similar to that mentioned above. In this structure, the cadmium cations are linked by pairs of thio­cyanate anions into chains that are either connected by two EtOH mol­ecules or urotropine ligands, which connect neighboring chains (FEWZOY; Barszcz *et al.*, 2013 ▸).

## Synthesis and crystallization

**Synthesis** Co(NCS)_2_ and urotropine were purchased from Merck. All chemicals were used without further purification.

Single crystals of **1** and **2** were obtained by reacting 0.15 mmol of Co(NCS)_2_ (26.3 mg) with 0.6 mmol of urotropine (84.1 mg) in 1 mL of ethanol after one day.

**Experimental details**

The data collection for single crystal structure analysis was performed using a Rigaku XtaLAB Synergy Dualflex kappa-diffractometer equipped with HyPix hybrid photon counting HPC detector, using Cu-Kα radiation from a PhotonJet micro-focus X-ray source.

The PXRD measurements were performed with Cu *K*α_1_ radiation (λ = 1.540598 Å) using a Stoe Transmission Powder Diffraction System (STADI P) equipped with a MYTHEN 1K detector and a Johansson-type Ge(111) monochromator.

## Refinement

Crystal data, data collection and structure refinement details are summarized in Table 5 ▸. All non-hydrogen atoms were refined anisotropically. The C—H hydrogen atoms were positioned with idealized geometry (methyl H atoms allowed to rotate but not to tip) and were refined isotropically with *U*_ĩso_(H) = 1.2*U*_eq_(C) (1.5 for methyl H atoms). The O—H hydrogen atoms were located in the difference map and were refined with restraints for the O—H distance (DFIX) and varying isotropic displacement parameters. The crystal of **1** was twinned by non-merohedry and therefore, a twin refinement using data in HKLF-5 format was performed where all equivalents were merged [BASF parameter = 0.309 (1)].

## Supplementary Material

Crystal structure: contains datablock(s) 1, 2. DOI: 10.1107/S2056989021013281/zv2011sup1.cif

Structure factors: contains datablock(s) 1. DOI: 10.1107/S2056989021013281/zv20111sup2.hkl

Structure factors: contains datablock(s) 2. DOI: 10.1107/S2056989021013281/zv20112sup3.hkl

Fig. S1. Calculated X-ray powder pattern for 1 (B) and 2 (C) as well as the experimental powder pattern of the crushed crystals (A). DOI: 10.1107/S2056989021013281/zv2011sup4.png

CCDC references: 2128606, 2128607

Additional supporting information:  crystallographic information; 3D view; checkCIF report

## Figures and Tables

**Table 1:** Selected bond lengths (Å) for **1** [Chem scheme1]

Co1—N1	2.037 (2)	Co1—O1	2.1620 (18)
Co1—N11	2.321 (2)		

**Table 2:** Selected bond lengths (Å) for **2** [Chem scheme1]

Co1—N1	2.078 (2)	Co1—O1	2.0894 (15)

**Table 3:** Hydrogen-bond geometry (Å, °) for **1** [Chem scheme1]

*D*—H⋯*A*	*D*—H	H⋯*A*	*D*⋯*A*	*D*—H⋯*A*
C13—H13*B*⋯S1^i^	0.99	2.81	3.781 (3)	168
C14—H14*A*⋯S1^ii^	0.99	2.98	3.816 (3)	143
C16—H16*A*⋯N1^iii^	0.99	2.57	3.166 (3)	119
C16—H16*B*⋯O1^iii^	0.99	2.49	3.090 (3)	119
O1—H1⋯N13^iv^	0.84 (2)	2.05 (3)	2.870 (3)	165 (4)
C22—H22*B*⋯S1^v^	0.98	3.02	3.989 (3)	169

Symmetry codes: (i) -x+{\script{3\over 2}}, y+{\script{1\over 2}}, -z+{\script{1\over 2}}; (ii) -x+{\script{1\over 2}}, y+{\script{1\over 2}}, -z+{\script{1\over 2}}; (iii) -x+1, -y+1, -z+1; (iv) x-1, y, z; (v) x, y+1, z.

**Table 4:** Hydrogen-bond geometry (Å, °) for **2** [Chem scheme1]

*D*—H⋯*A*	*D*—H	H⋯*A*	*D*⋯*A*	*D*—H⋯*A*
O1—H1⋯N11	0.87 (2)	1.95 (2)	2.799 (3)	163 (4)
C2—H2*B*⋯N1^i^	0.99	2.68	3.211 (3)	114

Symmetry code: (i) -x+1, -y+2, z.

**Table 5:** Experimental details

	**1**	**2**
Crystal data		
Chemical formula	[Co(NCS)_2_(C_6_H_12_N_4_)_2_(C_2_H_6_O)_2_]	[Co(NCS)_2_(C_2_H_6_O)_4_]·2C_6_H_12_N_4_
*M*_r_	547.62	499.56
Crystal system, space group	Monoclinic, *P*2_1_/*n*	Tetragonal, *P*\overline{4}*n*2
Temperature (K)	100	100
*a*, *b*, *c* (Å)	7.73205 (19), 11.5092 (3), 13.6693 (3)	9.69601 (6), 9.69601 (6), 12.94912 (14)
α, β, γ (°)	90, 95.376 (2), 90	90, 90, 90
*V* (Å^3^)	1211.08 (5)	1217.38 (2)
*Z*	2	2
Radiation type	Cu *K*α	Cu *K*α
μ (mm^−1^)	7.48	7.40
Crystal size (mm)	0.12 × 0.03 × 0.02	0.2 × 0.16 × 0.03

Data collection		
Diffractometer	XtaLAB Synergy, Dualflex, HyPix	XtaLAB Synergy, Dualflex, HyPix
Absorption correction	Multi-scan (*CrysAlis PRO*; Rigaku OD, 2021 ▸)	Multi-scan (*CrysAlis PRO*; Rigaku OD, 2021 ▸)
*T*_min_, *T*_max_	0.586, 1.000	0.786, 1.000
No. of measured, independent and observed [*I* > 2σ(*I*)] reflections	4731, 4731, 4504	34865, 1343, 1338
*R*_int_	–	0.030
(sin θ/λ)_max_ (Å^−1^)	0.639	0.639

Refinement		
*R*[*F* ^2^ > 2σ(*F* ^2^)], *wR*(*F* ^2^), *S*	0.044, 0.124, 1.06	0.024, 0.064, 1.08
No. of reflections	4731	1343
No. of parameters	157	75
No. of restraints	1	1
H-atom treatment	H atoms treated by a mixture of independent and constrained refinement	H atoms treated by a mixture of independent and constrained refinement
Δρ_max_, Δρ_min_ (e Å^−3^)	0.63, −0.43	0.20, −0.37
Absolute structure	–	F[(*I* ^+^)−(*I* ^−^)]/[(*I* ^+^)+(*I* ^−^)] lack *x* determined using 573 quotients (Parsons *et al.*, 2013 ▸)
Absolute structure parameter	–	0.0070 (19)

Computer programs: *CrysAlis PRO* (Rigaku OD, 2021 ▸), *SHELXT2014/5* (Sheldrick, 2015*a* ▸), *SHELXL2016/6* (Sheldrick, 2015*b* ▸), *DIAMOND* (Brandenburg & Putz, 1999 ▸) and *publCIF* (Westrip, 2010 ▸).

## supplementary crystallographic information

### Bis(ethanol-κO)bis(thiocyanato-κN)bis(urotropine-κN)cobalt(II) (1) Crystal data

**Table:**

[Co(NCS)_2_(C_6_H_12_N_4_)_2_(C_2_H_6_O)_2_]	*F*(000) = 578
*M**_r_* = 547.62	*D*_x_ = 1.502 Mg m^−^^3^
Monoclinic, *P*2_1_/*n*	Cu *K*α radiation, λ = 1.54184 Å
*a* = 7.73205 (19) Å	Cell parameters from 16954 reflections
*b* = 11.5092 (3) Å	θ = 5.0–79.3°
*c* = 13.6693 (3) Å	µ = 7.48 mm^−^^1^
β = 95.376 (2)°	*T* = 100 K
*V* = 1211.08 (5) Å^3^	Needle, light pink
*Z* = 2	0.12 × 0.03 × 0.02 mm

### Bis(ethanol-κO)bis(thiocyanato-κN)bis(urotropine-κN)cobalt(II) (1) Data collection

**Table:**

XtaLAB Synergy, Dualflex, HyPix diffractometer	4731 measured reflections
Radiation source: micro-focus sealed X-ray tube, PhotonJet (Cu) X-ray Source	4731 independent reflections
Mirror monochromator	4504 reflections with *I* > 2σ(*I*)
Detector resolution: 10.0000 pixels mm^-1^	θ_max_ = 80.3°, θ_min_ = 5.0°
ω scans	*h* = −9→9
Absorption correction: multi-scan (CrysAlisPro; Rigaku OD, 2021)	*k* = −14→14
*T*_min_ = 0.586, *T*_max_ = 1.000	*l* = −16→17

### Bis(ethanol-κO)bis(thiocyanato-κN)bis(urotropine-κN)cobalt(II) (1) Refinement

**Table:**

Refinement on *F*^2^	Primary atom site location: dual
Least-squares matrix: full	Hydrogen site location: mixed
*R*[*F*^2^ > 2σ(*F*^2^)] = 0.044	H atoms treated by a mixture of independent and constrained refinement
*wR*(*F*^2^) = 0.124	*w* = 1/[σ^2^(*F*_o_^2^) + (0.0719*P*)^2^ + 0.7955*P*] where *P* = (*F*_o_^2^ + 2*F*_c_^2^)/3
*S* = 1.06	(Δ/σ)_max_ < 0.001
4731 reflections	Δρ_max_ = 0.63 e Å^−^^3^
157 parameters	Δρ_min_ = −0.43 e Å^−^^3^
1 restraint	

### Bis(ethanol-κO)bis(thiocyanato-κN)bis(urotropine-κN)cobalt(II) (1) Special details

**Table:**

Geometry. All esds (except the esd in the dihedral angle between two l.s. planes) are estimated using the full covariance matrix. The cell esds are taken into account individually in the estimation of esds in distances, angles and torsion angles; correlations between esds in cell parameters are only used when they are defined by crystal symmetry. An approximate (isotropic) treatment of cell esds is used for estimating esds involving l.s. planes.
Refinement. Refined as a 2-component twin.

### Bis(ethanol-κO)bis(thiocyanato-κN)bis(urotropine-κN)cobalt(II) (1) Fractional atomic coordinates and isotropic or equivalent isotropic displacement parameters (Å^2^)

**Table:**

	*x*	*y*	*z*	*U*_iso_*/*U*_eq_	
Co1	0.500000	0.500000	0.500000	0.01842 (17)	
N1	0.3761 (3)	0.35322 (18)	0.44802 (17)	0.0228 (4)	
C1	0.3160 (3)	0.2624 (2)	0.43241 (18)	0.0227 (5)	
S1	0.23069 (10)	0.13371 (6)	0.41163 (5)	0.0324 (2)	
N11	0.6678 (3)	0.50619 (16)	0.36795 (16)	0.0187 (4)	
C11	0.6534 (3)	0.3991 (2)	0.30608 (19)	0.0225 (5)	
H11A	0.682796	0.330698	0.348296	0.027*	
H11B	0.531562	0.390244	0.277616	0.027*	
N12	0.7674 (3)	0.40116 (19)	0.22623 (16)	0.0240 (5)	
C12	0.9479 (4)	0.4157 (2)	0.2689 (2)	0.0244 (5)	
H12A	1.025654	0.417629	0.215328	0.029*	
H12B	0.981359	0.348202	0.311347	0.029*	
N13	0.9718 (3)	0.52416 (19)	0.32779 (16)	0.0216 (4)	
C13	0.9175 (3)	0.6229 (2)	0.2625 (2)	0.0226 (5)	
H13A	0.930800	0.696067	0.300510	0.027*	
H13B	0.994929	0.627096	0.208895	0.027*	
N14	0.7371 (3)	0.61247 (18)	0.21969 (16)	0.0211 (4)	
C14	0.6256 (3)	0.6071 (2)	0.30005 (19)	0.0203 (5)	
H14A	0.503020	0.600855	0.272245	0.024*	
H14B	0.637958	0.680151	0.338260	0.024*	
C15	0.7207 (4)	0.5030 (2)	0.1637 (2)	0.0244 (6)	
H15A	0.797350	0.505963	0.109629	0.029*	
H15B	0.599568	0.494447	0.134039	0.029*	
C16	0.8536 (3)	0.5178 (2)	0.40565 (19)	0.0199 (5)	
H16A	0.867948	0.588774	0.446484	0.024*	
H16B	0.886778	0.450435	0.448420	0.024*	
O1	0.2998 (2)	0.60272 (15)	0.41991 (14)	0.0217 (4)	
H1	0.215 (4)	0.570 (4)	0.389 (3)	0.058 (13)*	
C21	0.2958 (3)	0.7251 (2)	0.3965 (2)	0.0248 (5)	
H21A	0.257744	0.734961	0.325847	0.030*	
H21B	0.414779	0.757096	0.408512	0.030*	
C22	0.1752 (4)	0.7938 (2)	0.4565 (2)	0.0287 (6)	
H22A	0.056867	0.763078	0.444535	0.043*	
H22B	0.176250	0.875727	0.437066	0.043*	
H22C	0.214619	0.786813	0.526478	0.043*	

### Bis(ethanol-κO)bis(thiocyanato-κN)bis(urotropine-κN)cobalt(II) (1) Atomic displacement parameters (Å^2^)

**Table:**

	*U*^11^	*U*^22^	*U*^33^	*U*^12^	*U*^13^	*U*^23^
Co1	0.0184 (3)	0.0121 (3)	0.0244 (3)	−0.00104 (19)	0.0005 (2)	−0.0001 (2)
N1	0.0221 (10)	0.0161 (10)	0.0303 (11)	−0.0029 (8)	0.0037 (8)	−0.0025 (8)
C1	0.0216 (11)	0.0232 (12)	0.0236 (11)	0.0011 (9)	0.0038 (9)	−0.0023 (10)
S1	0.0378 (4)	0.0239 (3)	0.0362 (4)	−0.0115 (3)	0.0076 (3)	−0.0050 (3)
N11	0.0193 (10)	0.0150 (10)	0.0215 (10)	−0.0013 (7)	0.0006 (8)	0.0007 (7)
C11	0.0262 (12)	0.0160 (11)	0.0250 (12)	−0.0013 (9)	0.0008 (10)	−0.0009 (10)
N12	0.0314 (12)	0.0179 (10)	0.0229 (10)	0.0009 (8)	0.0031 (9)	−0.0016 (8)
C12	0.0269 (13)	0.0199 (12)	0.0270 (13)	0.0040 (10)	0.0050 (10)	0.0012 (10)
N13	0.0219 (10)	0.0193 (10)	0.0239 (10)	0.0014 (8)	0.0028 (8)	0.0016 (9)
C13	0.0220 (12)	0.0196 (11)	0.0264 (13)	−0.0003 (9)	0.0034 (10)	0.0031 (10)
N14	0.0235 (10)	0.0172 (9)	0.0225 (10)	0.0006 (8)	0.0013 (8)	0.0019 (8)
C14	0.0214 (11)	0.0153 (11)	0.0240 (12)	0.0018 (9)	0.0007 (10)	0.0010 (9)
C15	0.0303 (14)	0.0210 (13)	0.0216 (13)	0.0005 (9)	0.0006 (11)	0.0007 (9)
C16	0.0194 (11)	0.0181 (10)	0.0221 (12)	0.0001 (9)	0.0009 (9)	0.0016 (9)
O1	0.0210 (8)	0.0143 (8)	0.0297 (9)	0.0002 (6)	0.0009 (7)	0.0034 (7)
C21	0.0248 (12)	0.0171 (11)	0.0328 (13)	0.0001 (9)	0.0036 (10)	0.0033 (10)
C22	0.0333 (14)	0.0217 (12)	0.0305 (13)	0.0027 (10)	0.0005 (11)	−0.0029 (11)

### Bis(ethanol-κO)bis(thiocyanato-κN)bis(urotropine-κN)cobalt(II) (1) Geometric parameters (Å, º)

**Table:**

Co1—N1^i^	2.037 (2)	N13—C16	1.468 (3)
Co1—N1	2.037 (2)	C13—H13A	0.9900
Co1—N11^i^	2.321 (2)	C13—H13B	0.9900
Co1—N11	2.321 (2)	C13—N14	1.466 (3)
Co1—O1	2.1620 (18)	N14—C14	1.460 (3)
Co1—O1^i^	2.1620 (18)	N14—C15	1.474 (3)
N1—C1	1.156 (3)	C14—H14A	0.9900
C1—S1	1.635 (3)	C14—H14B	0.9900
N11—C11	1.493 (3)	C15—H15A	0.9900
N11—C14	1.503 (3)	C15—H15B	0.9900
N11—C16	1.486 (3)	C16—H16A	0.9900
C11—H11A	0.9900	C16—H16B	0.9900
C11—H11B	0.9900	O1—H1	0.84 (2)
C11—N12	1.466 (3)	O1—C21	1.444 (3)
N12—C12	1.471 (4)	C21—H21A	0.9900
N12—C15	1.475 (3)	C21—H21B	0.9900
C12—H12A	0.9900	C21—C22	1.519 (4)
C12—H12B	0.9900	C22—H22A	0.9800
C12—N13	1.487 (3)	C22—H22B	0.9800
N13—C13	1.481 (3)	C22—H22C	0.9800
			
N1^i^—Co1—N1	180.0	N13—C13—H13B	109.1
N1—Co1—N11	91.89 (8)	H13A—C13—H13B	107.8
N1^i^—Co1—N11^i^	91.89 (8)	N14—C13—N13	112.5 (2)
N1^i^—Co1—N11	88.11 (8)	N14—C13—H13A	109.1
N1—Co1—N11^i^	88.11 (8)	N14—C13—H13B	109.1
N1^i^—Co1—O1^i^	89.19 (8)	C13—N14—C15	108.0 (2)
N1—Co1—O1	89.19 (8)	C14—N14—C13	108.1 (2)
N1—Co1—O1^i^	90.81 (8)	C14—N14—C15	109.0 (2)
N1^i^—Co1—O1	90.81 (8)	N11—C14—H14A	109.0
N11^i^—Co1—N11	180.0	N11—C14—H14B	109.0
O1—Co1—N11	90.87 (7)	N14—C14—N11	112.8 (2)
O1^i^—Co1—N11	89.13 (7)	N14—C14—H14A	109.0
O1^i^—Co1—N11^i^	90.87 (7)	N14—C14—H14B	109.0
O1—Co1—N11^i^	89.13 (7)	H14A—C14—H14B	107.8
O1^i^—Co1—O1	180.0	N12—C15—H15A	109.2
C1—N1—Co1	169.3 (2)	N12—C15—H15B	109.2
N1—C1—S1	179.4 (3)	N14—C15—N12	111.9 (2)
C11—N11—Co1	113.44 (15)	N14—C15—H15A	109.2
C11—N11—C14	106.7 (2)	N14—C15—H15B	109.2
C14—N11—Co1	113.53 (15)	H15A—C15—H15B	107.9
C16—N11—Co1	109.01 (16)	N11—C16—H16A	108.8
C16—N11—C11	106.79 (19)	N11—C16—H16B	108.8
C16—N11—C14	106.96 (19)	N13—C16—N11	113.6 (2)
N11—C11—H11A	109.0	N13—C16—H16A	108.8
N11—C11—H11B	109.0	N13—C16—H16B	108.8
H11A—C11—H11B	107.8	H16A—C16—H16B	107.7
N12—C11—N11	113.0 (2)	Co1—O1—H1	120 (3)
N12—C11—H11A	109.0	C21—O1—Co1	130.16 (16)
N12—C11—H11B	109.0	C21—O1—H1	109 (3)
C11—N12—C12	108.7 (2)	O1—C21—H21A	109.0
C11—N12—C15	108.2 (2)	O1—C21—H21B	109.0
C12—N12—C15	108.1 (2)	O1—C21—C22	112.9 (2)
N12—C12—H12A	109.2	H21A—C21—H21B	107.8
N12—C12—H12B	109.2	C22—C21—H21A	109.0
N12—C12—N13	112.0 (2)	C22—C21—H21B	109.0
H12A—C12—H12B	107.9	C21—C22—H22A	109.5
N13—C12—H12A	109.2	C21—C22—H22B	109.5
N13—C12—H12B	109.2	C21—C22—H22C	109.5
C13—N13—C12	107.7 (2)	H22A—C22—H22B	109.5
C16—N13—C12	107.3 (2)	H22A—C22—H22C	109.5
C16—N13—C13	108.4 (2)	H22B—C22—H22C	109.5
N13—C13—H13A	109.1		
			
Co1—N11—C11—N12	−176.71 (16)	C12—N13—C16—N11	−59.2 (3)
Co1—N11—C14—N14	177.58 (15)	N13—C13—N14—C14	59.1 (3)
Co1—N11—C16—N13	−179.20 (15)	N13—C13—N14—C15	−58.7 (3)
Co1—O1—C21—C22	−107.1 (2)	C13—N13—C16—N11	56.8 (3)
N11—C11—N12—C12	58.0 (3)	C13—N14—C14—N11	−59.3 (3)
N11—C11—N12—C15	−59.2 (3)	C13—N14—C15—N12	59.0 (3)
C11—N11—C14—N14	−56.7 (3)	C14—N11—C11—N12	57.5 (3)
C11—N11—C16—N13	57.9 (3)	C14—N11—C16—N13	−56.1 (3)
C11—N12—C12—N13	−58.6 (3)	C14—N14—C15—N12	−58.2 (3)
C11—N12—C15—N14	58.4 (3)	C15—N12—C12—N13	58.7 (3)
N12—C12—N13—C13	−57.9 (3)	C15—N14—C14—N11	57.9 (3)
N12—C12—N13—C16	58.6 (3)	C16—N11—C11—N12	−56.6 (3)
C12—N12—C15—N14	−59.2 (3)	C16—N11—C14—N14	57.3 (3)
C12—N13—C13—N14	58.0 (3)	C16—N13—C13—N14	−57.7 (3)

Symmetry code: (i) −*x*+1, −*y*+1, −*z*+1.

### Bis(ethanol-κO)bis(thiocyanato-κN)bis(urotropine-κN)cobalt(II) (1) Hydrogen-bond geometry (Å, º)

**Table:**

*D*—H···*A*	*D*—H	H···*A*	*D*···*A*	*D*—H···*A*
C13—H13*B*···S1^ii^	0.99	2.81	3.781 (3)	168
C14—H14*A*···S1^iii^	0.99	2.98	3.816 (3)	143
C16—H16*A*···N1^i^	0.99	2.57	3.166 (3)	119
C16—H16*B*···O1^i^	0.99	2.49	3.090 (3)	119
O1—H1···N13^iv^	0.84 (2)	2.05 (3)	2.870 (3)	165 (4)
C22—H22*B*···S1^v^	0.98	3.02	3.989 (3)	169

Symmetry codes: (i) −*x*+1, −*y*+1, −*z*+1; (ii) −*x*+3/2, *y*+1/2, −*z*+1/2; (iii) −*x*+1/2, *y*+1/2, −*z*+1/2; (iv) *x*−1, *y*, *z*; (v) *x*, *y*+1, *z*.

### Tetrakis(ethanol-κO)bis(thiocyanato-κN)cobalt(II)–urotropine (1/2) (2) Crystal data

**Table:**

[Co(NCS)_2_(C_2_H_6_O)_4_]·2C_6_H_12_N_4_	*D*_x_ = 1.363 Mg m^−^^3^
*M**_r_* = 499.56	Cu *K*α radiation, λ = 1.54184 Å
Tetragonal, *P*4*n*2	Cell parameters from 26612 reflections
*a* = 9.69601 (6) Å	θ = 5.7–79.2°
*c* = 12.94912 (14) Å	µ = 7.40 mm^−^^1^
*V* = 1217.38 (2) Å^3^	*T* = 100 K
*Z* = 2	Plate, light violet
*F*(000) = 530	0.2 × 0.16 × 0.03 mm

### Tetrakis(ethanol-κO)bis(thiocyanato-κN)cobalt(II)–urotropine (1/2) (2) Data collection

**Table:**

XtaLAB Synergy, Dualflex, HyPix diffractometer	1343 independent reflections
Radiation source: micro-focus sealed X-ray tube, PhotonJet (Cu) X-ray Source	1338 reflections with *I* > 2σ(*I*)
Mirror monochromator	*R*_int_ = 0.030
Detector resolution: 10.0000 pixels mm^-1^	θ_max_ = 80.1°, θ_min_ = 5.7°
ω scans	*h* = −12→11
Absorption correction: multi-scan (CrysAlisPro; Rigaku OD, 2021)	*k* = −12→12
*T*_min_ = 0.786, *T*_max_ = 1.000	*l* = −16→16
34865 measured reflections	

### Tetrakis(ethanol-κO)bis(thiocyanato-κN)cobalt(II)–urotropine (1/2) (2) Refinement

**Table:**

Refinement on *F*^2^	H atoms treated by a mixture of independent and constrained refinement
Least-squares matrix: full	*w* = 1/[σ^2^(*F*_o_^2^) + (0.0326*P*)^2^ + 0.587*P*] where *P* = (*F*_o_^2^ + 2*F*_c_^2^)/3
*R*[*F*^2^ > 2σ(*F*^2^)] = 0.024	(Δ/σ)_max_ < 0.001
*wR*(*F*^2^) = 0.064	Δρ_max_ = 0.20 e Å^−^^3^
*S* = 1.08	Δρ_min_ = −0.37 e Å^−^^3^
1343 reflections	Extinction correction: SHELXL-2016/6 (Sheldrick 2016), Fc^*^=kFc[1+0.001xFc^2^λ^3^/sin(2θ)]^-1/4^
75 parameters	Extinction coefficient: 0.0019 (4)
1 restraint	Absolute structure: F[(*I*^+^)-(*I*^-^)]/[(*I*^+^)+(*I*^-^)] lack *x* determined using 573 quotients(Parsons *et al.*, 2013)
Primary atom site location: dual	Absolute structure parameter: 0.0070 (19)
Hydrogen site location: mixed	

### Tetrakis(ethanol-κO)bis(thiocyanato-κN)cobalt(II)–urotropine (1/2) (2) Special details

**Table:**

Geometry. All esds (except the esd in the dihedral angle between two l.s. planes) are estimated using the full covariance matrix. The cell esds are taken into account individually in the estimation of esds in distances, angles and torsion angles; correlations between esds in cell parameters are only used when they are defined by crystal symmetry. An approximate (isotropic) treatment of cell esds is used for estimating esds involving l.s. planes.

### Tetrakis(ethanol-κO)bis(thiocyanato-κN)cobalt(II)–urotropine (1/2) (2) Fractional atomic coordinates and isotropic or equivalent isotropic displacement parameters (Å^2^)

**Table:**

	*x*	*y*	*z*	*U*_iso_*/*U*_eq_	Occ. (<1)
Co1	0.500000	1.000000	0.750000	0.01833 (18)	
N1	0.34847 (17)	0.84847 (17)	0.750000	0.0233 (5)	
C1	0.2634 (2)	0.7634 (2)	0.750000	0.0212 (5)	
S1	0.14431 (5)	0.64431 (5)	0.750000	0.0340 (2)	
O1	0.5977 (2)	0.88742 (18)	0.86652 (13)	0.0275 (4)	
H1	0.587 (4)	0.799 (2)	0.875 (3)	0.048 (10)*	
C2	0.7177 (3)	0.9247 (3)	0.9237 (2)	0.0361 (6)	
H2A	0.709202	0.890137	0.995411	0.043*	
H2B	0.725516	1.026420	0.926484	0.043*	
C3	0.8444 (3)	0.8658 (4)	0.8752 (3)	0.0557 (10)	
H3A	0.852958	0.900109	0.804352	0.084*	
H3B	0.837857	0.764901	0.874324	0.084*	
H3C	0.925615	0.893581	0.915212	0.084*	
N11	0.54520 (19)	0.61785 (19)	0.93312 (14)	0.0186 (4)	
C11	0.500000	0.500000	0.8688 (2)	0.0197 (6)	
H11A	0.577109	0.470426	0.823830	0.024*	0.5
H11B	0.422888	0.529574	0.823832	0.024*	0.5
C12	0.6597 (2)	0.5713 (2)	0.99965 (16)	0.0195 (4)	
H12A	0.690997	0.649175	1.043227	0.023*	
H12B	0.738159	0.542300	0.955816	0.023*	

### Tetrakis(ethanol-κO)bis(thiocyanato-κN)cobalt(II)–urotropine (1/2) (2) Atomic displacement parameters (Å^2^)

**Table:**

	*U*^11^	*U*^22^	*U*^33^	*U*^12^	*U*^13^	*U*^23^
Co1	0.0176 (2)	0.0176 (2)	0.0198 (3)	−0.0025 (2)	0.000	0.000
N1	0.0229 (7)	0.0229 (7)	0.0241 (11)	−0.0033 (9)	0.0000 (9)	0.0000 (9)
C1	0.0203 (8)	0.0203 (8)	0.0230 (12)	0.0031 (10)	−0.0012 (10)	0.0012 (10)
S1	0.0265 (3)	0.0265 (3)	0.0488 (5)	−0.0090 (3)	−0.0070 (3)	0.0070 (3)
O1	0.0293 (9)	0.0198 (8)	0.0332 (8)	−0.0061 (6)	−0.0102 (7)	0.0058 (7)
C2	0.0442 (16)	0.0289 (12)	0.0352 (13)	−0.0127 (11)	−0.0183 (12)	0.0089 (11)
C3	0.0286 (14)	0.074 (2)	0.064 (2)	−0.0106 (15)	−0.0070 (15)	0.0378 (19)
N11	0.0164 (8)	0.0185 (8)	0.0210 (8)	0.0007 (7)	0.0006 (7)	0.0021 (7)
C11	0.0193 (14)	0.0198 (14)	0.0199 (14)	0.0005 (11)	0.000	0.000
C12	0.0164 (10)	0.0194 (11)	0.0226 (10)	−0.0016 (8)	−0.0021 (8)	0.0016 (8)

### Tetrakis(ethanol-κO)bis(thiocyanato-κN)cobalt(II)–urotropine (1/2) (2) Geometric parameters (Å, º)

**Table:**

Co1—N1^i^	2.078 (2)	C2—C3	1.494 (5)
Co1—N1	2.078 (2)	C3—H3A	0.9800
Co1—O1	2.0894 (15)	C3—H3B	0.9800
Co1—O1^i^	2.0894 (15)	C3—H3C	0.9800
Co1—O1^ii^	2.0894 (15)	N11—C11	1.481 (2)
Co1—O1^iii^	2.0894 (15)	N11—C12^iv^	1.483 (3)
N1—C1	1.166 (4)	N11—C12	1.476 (3)
C1—S1	1.633 (3)	C11—H11A	0.9900
O1—H1	0.87 (2)	C11—H11B	0.9900
O1—C2	1.426 (3)	C12—H12A	0.9900
C2—H2A	0.9900	C12—H12B	0.9900
C2—H2B	0.9900		
			
N1^i^—Co1—N1	180.0	C3—C2—H2A	109.5
N1—Co1—O1^i^	92.80 (6)	C3—C2—H2B	109.5
N1—Co1—O1^iii^	92.80 (6)	C2—C3—H3A	109.5
N1^i^—Co1—O1^ii^	92.80 (6)	C2—C3—H3B	109.5
N1^i^—Co1—O1^iii^	87.20 (6)	C2—C3—H3C	109.5
N1—Co1—O1^ii^	87.20 (6)	H3A—C3—H3B	109.5
N1—Co1—O1	87.20 (6)	H3A—C3—H3C	109.5
N1^i^—Co1—O1^i^	87.20 (6)	H3B—C3—H3C	109.5
N1^i^—Co1—O1	92.80 (6)	C11—N11—C12^iv^	108.42 (15)
O1^i^—Co1—O1^iii^	174.40 (11)	C12—N11—C11	108.38 (16)
O1^ii^—Co1—O1^iii^	87.54 (9)	C12—N11—C12^iv^	108.31 (13)
O1^i^—Co1—O1	87.53 (9)	N11—C11—N11^v^	111.5 (2)
O1^ii^—Co1—O1^i^	92.74 (9)	N11^v^—C11—H11A	109.3
O1^ii^—Co1—O1	174.40 (11)	N11—C11—H11A	109.3
O1^iii^—Co1—O1	92.74 (10)	N11—C11—H11B	109.3
C1—N1—Co1	180.00 (18)	N11^v^—C11—H11B	109.3
N1—C1—S1	180.0 (2)	H11A—C11—H11B	108.0
Co1—O1—H1	123 (2)	N11—C12—N11^vi^	111.70 (19)
C2—O1—Co1	127.80 (16)	N11—C12—H12A	109.3
C2—O1—H1	107 (2)	N11^vi^—C12—H12A	109.3
O1—C2—H2A	109.5	N11—C12—H12B	109.3
O1—C2—H2B	109.5	N11^vi^—C12—H12B	109.3
O1—C2—C3	110.8 (3)	H12A—C12—H12B	107.9
H2A—C2—H2B	108.1		
			
Co1—O1—C2—C3	−94.0 (2)	C12^iv^—N11—C11—N11^v^	−58.55 (12)
C11—N11—C12—N11^vi^	−58.9 (2)	C12^iv^—N11—C12—N11^vi^	58.52 (16)
C12—N11—C11—N11^v^	58.80 (12)		

Symmetry codes: (i) −*x*+1, −*y*+2, *z*; (ii) *y*−1/2, *x*+1/2, −*z*+3/2; (iii) −*y*+3/2, −*x*+3/2, −*z*+3/2; (iv) −*y*+1, *x*, −*z*+2; (v) −*x*+1, −*y*+1, *z*; (vi) *y*, −*x*+1, −*z*+2.

### Tetrakis(ethanol-κO)bis(thiocyanato-κN)cobalt(II)–urotropine (1/2) (2) Hydrogen-bond geometry (Å, º)

**Table:**

*D*—H···*A*	*D*—H	H···*A*	*D*···*A*	*D*—H···*A*
O1—H1···N11	0.87 (2)	1.95 (2)	2.799 (3)	163 (4)
C2—H2*B*···N1^i^	0.99	2.68	3.211 (3)	114

Symmetry code: (i) −*x*+1, −*y*+2, *z*.
