# Supplementary figures and images for: Crystal structure of di­ethano­lbis(thio­cyanato)­bis(urotropine)cobalt(II) and tetra­ethano­lbis(thio­cyanato)­cobalt(II)–urotropine (1/2)
Source: Acta Crystallogr E Crystallogr Commun. 2022 Jan 1;78(Pt 1):66–70. doi: 10.1107/S2056989021013281 (PMC8739191; doi:10.1107/S2056989021013281)

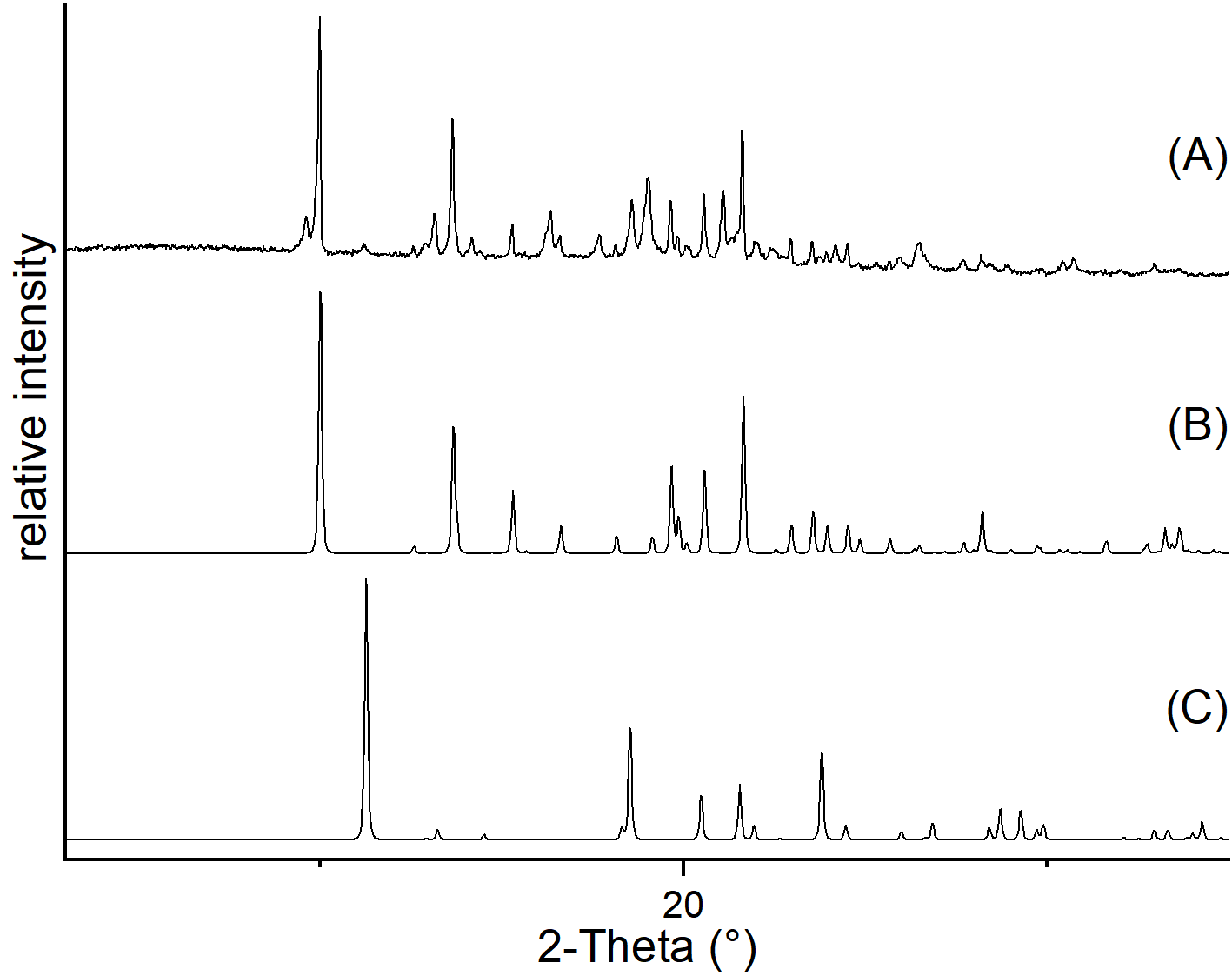

Supplement: Supplementary file 4 [file e-78-00066-sup4.png]
